# HIF1α/TET1 Pathway Mediates Hypoxia-Induced Adipocytokine Promoter Hypomethylation in Human Adipocytes

**DOI:** 10.3390/cells9010134

**Published:** 2020-01-06

**Authors:** Mohamed M. Ali, Shane A. Phillips, Abeer M. Mahmoud

**Affiliations:** 1 Department of Physical Therapy and Integrative Physiology Laboratory, College of Applied Health Sciences, University of Illinois at Chicago, Chicago, IL 60612, USA; mali37@uic.edu; 2 Department of Physical Therapy and Integrative Physiology Laboratory, College of Applied Health Sciences and Division of Endocrinology, Diabetes, and Metabolism, University of Illinois at Chicago, Chicago, IL 60612, USA; shanep@uic.edu; 3 Department of Medicine, College of Medicine, University of Illinois at Chicago, Chicago, IL 60612, USA

**Keywords:** adipocytes, hypoxia, inflammation, DNA methylation, HIF1α, TET1, adipocytokines, epigenetics

## Abstract

Obesity is associated with the accumulation of dysfunctional adipose tissue that secretes several pro-inflammatory cytokines (adipocytokines). Recent studies have presented evidence that adipose tissues in obese individuals and animal models are hypoxic, which may result in upregulation and stabilization of the hypoxia inducible factor HIF1α. Epigenetic mechanisms such as DNA methylation enable the body to respond to microenvironmental changes such as hypoxia and may represent a mechanistic link between obesity-associated hypoxia and upregulated inflammatory adipocytokines. The purpose of this study was to investigate the role of hypoxia in modifying adipocytokine DNA methylation and subsequently adipocytokine expression. We suggested that this mechanism is mediated via the DNA demethylase, ten-eleven translocation-1 (TET1), transcription of which has been shown to be induced by HIF1α. To this end, we studied the effect of hypoxia (2% O_2_) in differentiated subcutaneous human adipocytes in the presence or absence of HIF1α stabilizer (Dimethyloxalylglycine (DMOG), 500 μM), HIF1α inhibitor (methyl 3-[[2-[4-(2-adamantyl) phenoxy] acetyl] amino]-4-hydroxybenzoate, 30 μM), or TET1-specific siRNA. Subjecting the adipocytes to hypoxia significantly induced HIF1α and TET1 protein levels. Moreover, hypoxia induced global hydroxymethylation, reduced adipocytokine DNA promoter methylation, and induced adipocytokine expression. These effects were abolished by either HIF1α inhibitor or TET1 gene silencing. The major hypoxia-responsive adipocytokines were leptin, interleukin-1 (IL6), IL1β, tumor necrosis factor α (TNFα), and interferon γ (IFNγ). Overall, these data demonstrate an activation of the hydroxymethylation pathway mediated by TET1. This pathway contributes to promoter hypomethylation and gene upregulation of the inflammatory adipocytokines in adipocytes in response to hypoxia.

## 1. Introduction

Obesity is a common and serious disease that affects more than one third (36.5%) of U.S. adults and over 600 million adults worldwide [1]. The most common preventable leading causes of death in obese adults are cardiovascular (CVD), diabetes, and cancer [2]. Thus, unlocking the complicated interplay between obesity and its co-morbidities by investigating the changes it causes in gene expression is extremely significant. Obesity is characterized by a large accumulation of dysfunctional adipose tissue, which is now recognized as an endocrine organ that secretes numerous hormones and inflammatory cytokines, generating a systemic proinflammatory state [3]. Obesity is also associated with hypoxia in adipose tissues [4]. Our current hypothesis is that hypoxia in adipose tissues triggers changes in the DNA methylation profile in adipocytes. DNA methylation is an epigenetic mechanism that is essential for regulating gene transcription. However, aberrant DNA hyper- or hypomethylation can result in unscheduled gene overexpression. Aberrant DNA methylation can be induced by changes in the surrounding microenvironment such as oxygen levels [5]. Previous studies suggested that hypoxia modifies DNA methylation via interfering with DNA methyl transferase (DNMT) activity and the bioavailability of its methyl donor, S-adenosyl methionine (SAM) [6,7]. Recently, it has been shown that DNA hypomethylation in cancer can be induced via active demethylation by the hydroxy methylase enzymes, TETs [8,9]. In the current study, we hypothesize that DNA hypomethylation that accompanies hypoxia may occur secondary to an increased expression of the HIF-1α-inducible TET1 enzyme. TET1 enzyme oxidizes 5- methylcytosine (5-mC) to 5-hydroxy-mC (5-hmC), which is modified through several suggested mechanisms including deamination and decarboxylation that ultimately lead to base excision repair and replacement with an unmethylated cytosine [10]. TET1 is the most prominent member of the TET family, and previous studies showed that the knockdown of TET1 results in increased global methylation in mice [11]. Efforts are underway to study the role of TET1 in cancer and developmental diseases, however there is an obvious lack in studies investigating this mechanism in the context of obesity [12]. The current study is expected to advance our knowledge gap and to elucidate DNA hypomethylation as an underlying epigenetic mechanism for the uncontrolled production of inflammatory adipocytokines under conditions of tissue hypoxia and identify the intracellular signal, HIF1α/TET1, as an upstream target that initiates DNA hypomethylation.

## 2. Materials and Methods

### 2.1. Chemical and Reagents

Dimethyloxalylglycine (DMOG) and 5-Aza- 2′deoxycytidine (5-Aza-dC) were purchased from Sigma-Aldrich (St. Louis, MO, USA). Methyl 3-[[2-[4-(2-adamantyl) phenoxy] acetyl] amino]-4-hydroxybenzoate (CAY10585) was purchased from Abcam (Cambridge, MA, USA). S-adenosylmethionine (SAM) was purchased from Santa Cruz Biotechnology (Santa Cruz, CA, USA).

### 2.2. Culture of Primary Subcutaneous Pre-Adipocytes

Human pre-adipocytes were purchased from ScienCell Research Laboratories (Carlsbad, CA, USA) where they were characterized by immunofluorescence with antibodies specific to CD24, CD44, CD90 and lipid staining after differentiation. Preadipocytes were cultured in optimized Fibroblast Growth Low Serum Medium (ATCC, VA, USA) containing 5 ng/mL FGF β, 7.5 mM l-glutamine, 50 μg/mL ascorbic acid, 1 μg/mL hydrocortisone hemisuccinate, 5 μg/mL insulin, and 2% fetal bovine serum. Cells were maintained at 37 °C in a humidified atmosphere and differentiated to mature adipocytes using an Adipocyte Differentiation Toolkit that consists of preadipocyte growth medium, adipocyte differentiation initiation medium, and adipocyte differentiation maintenance medium, all purchased from ScienCell Research Laboratories. First, preadipocytes were cultured for 48 h in preadipocyte growth medium which contains Dulbecco’s Modified Eagle Medium (DMEM)/Ham’s F-12 (1:1, *v*/*v*), 4-(2-hydroxyethyl)-1-piperazineethanesulfonic acid (HEPES) pH 7.4, 5% fetal bovine serum (FBS), penicillin, streptomycin, and amphotericin B. Cells were then incubated in Adipocyte Differentiation Initiation Medium for 48 h. This medium consists of DMEM/Ham’s F-12 (1:1, *v*/*v*), HEPES pH 7.4, 5% FBS, biotin, pantothenate, human insulin, dexamethasone 3-isobutyl-1-methylxanthine (IBMX) peroxisome proliferator-activated receptor gamma (PPARγ) agonist, penicillin, streptomycin, and amphotericin B. Finally, cells were maintained in differentiation maintenance medium for 12–14 days. This medium consists of DMEM/Ham’s F-12 (1:1, *v*/*v*), HEPES pH 7.4, 5% FBS, biotin, pantothenate, human insulin, dexamethasone, penicillin, streptomycin, and amphotericin B. To confirm maturation, cells were fixed and stained with Oil Red O stain.

### 2.3. Oil Red O Staining

**Figure 1:**
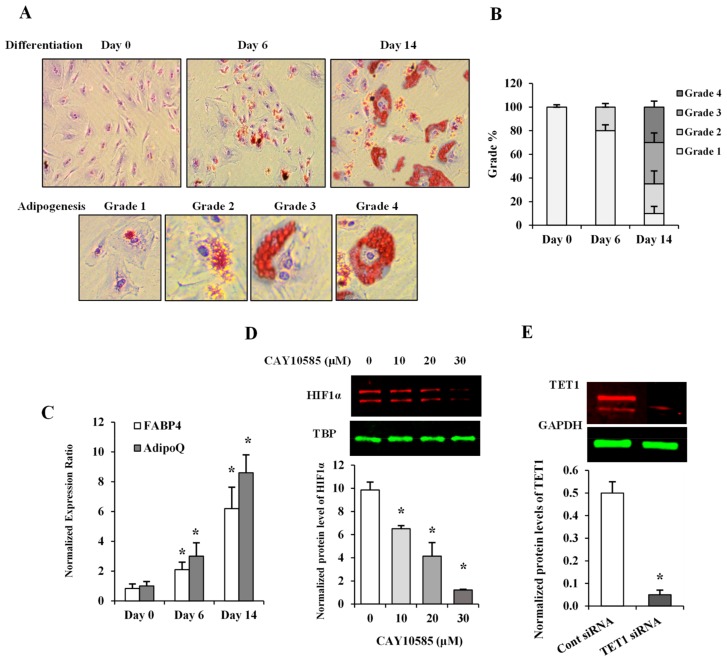
Assessment of adipocyte differentiation and HIF1α and TET1 inhibition in differentiated adipocytes. (**A**) Microscopic grading of adipocyte differentiation based on cytoplasmic accumulation of lipid droplets by differentiation days 0, 6, and 14 (upper panel; original magnification 10X). Representative images of individual cells with varying degrees of lipid accumulation that reflect different grades of adipogenesis (lower panel; original magnification 20X). (**B**) A stacked column chart representing the percentage of cells assigned to grades 1-4 in differentiating adipocytes at days 0, 6, and 14. (**C**) Quantitative assessment by real-time PCR of mRNA levels of FABP4 and AdipoQ normalized to the house keeping gene, β-actin. (**D**,**E**) Western blot analysis and quantification of the normalized signal intensity of HIF1α protein in response to increasing doses of the HIF1α inhibitor, CAY10585 (0–30 µM) for 24 h (**D**) and TET1 protein after incubation with a pool of three target-specific TET1 siRNAs for 7 h (**E**). Results represent the means ± SD for three independent experiments in triplicates for each treatment (n = 9), (* *p* < 0.05) for comparison with control.

Lipid droplets within cultured cells were detected using (Oil Red O) Staining Kit (BioVision Inc., Milpitas, CA, USA). Briefly, cells were washed with 1X PBS, fixed in 10% formalin for 60 min and washed twice with dH_2_O. Cells were then incubated with 60% isopropanol for 5 min followed by an incubation in Oil Red O Stain for 10 min. The stain was removed, and cells were washed with dH_2_O 2–5 times or until excess stain is no longer apparent. This was followed by incubation with hematoxylin for 60 s followed by washing several times until excess hematoxylin is completely removed. Cells were then kept covered with dH_2_O and viewed under an inverted microscope (Olympus, Shinjuku, Tokyo, Japan). The amount of fat droplets in individual cells was scored following a grading scheme developed by English et al. [13]. Briefly, the level of adipogenesis was assessed in 500 cells and classified based on the proportion of cytoplasm occupied by fat globules into: grade 1: 0–24%; grade 2: 25–49%; grade 3: 50–74%; and grade 4: 75–100% (Figure 1A). Subsequently, a corresponding percentage of cells at each grade was calculated. All experiments were performed in triplicates and averaged.

### 2.4. Induction of Hypoxia in Differentiated Adipocytes

For hypoxia treatment, differentiated adipocytes were cultured in 2% O_2_ and 5% CO_2_ with or without 500 µmol/L of the HIF1α stabilizer, DMOG, in Heracell CO_2_ incubator with adjustable O_2_ tension (1–21%) (Thermo Fisher Scientific, Waltham, MA, USA) at 37 °C and compared to control cells cultured under normoxia (20% O_2_ and 5% CO_2_) for 24 h. To attenuate HIF1α transcriptional activity, cells were treated with CAY10585 at concentrations of 10, 20, or 30 μM for 24 h followed by analyses of the nuclear fraction of HIF1α protein.

### 2.5. TET1 Gene Silencing in Mature Adipocytes

Mature adipocytes were seeded into 6-well plate at a concentration of 2 × 10^5^ cells per well in 2 mL Fibroblast Growth Low Serum Medium and incubated at 37 °C and 5% CO_2_. Twenty-four h later, cells were transiently transfected with small interfering RNAs (siRNAs) pool that consists of three target-specific 19–25 nt siRNAs designed for effective knockdown of the TET1 gene (Santa Cruz Biotechnology). Transfection was performed following the standard protocol. Briefly, 1µg of either the scrambled control siRNA or TET1 siRNA was used in a total volume of 1 mL transfection media in which cells were incubated for 7 h. At the end of incubation period, transfection media were replaced with fresh growth media. Validation of TET1 knock-down was performed by western blot analysis of the total protein that was collected from transfected cells using a rabbit polyclonal TET1 antibody (ab191698) from Abcam.

### 2.6. Real-Time Polymerase Chain Reaction (PCR)

Total RNA was extracted using RNeasy mini kits (Qiagen, Germantown, MD, USA). RNA quantity and quality were determined via spectrophotometer. Total RNA (5 μg) was reverse transcribed into cDNA using SuperScript RT III (Invitrogen). Gene expression was determined via real-time RT-PCR using SYBR Green (Applied Biosystems, Foster, CA, USA) and custom designed primers for Primers for leptin, IL1β, IL6, IL8, IL17, C-X-C Motif Chemokine Ligand 5 (CXCL5), macrophage migration inhibitory factor (MIF), vascular endothelial growth factor (VEGF), TNF-α, IFNγ, PDK1 (pyruvate dehydrogenase kinase 1), FABP4 (fatty acids binding protein 4), and Adopnectin (AdipoQ) genes were designed using primer3 software v. 0.4.0 and manufactured by Invitrogen Life Technologies (Table 1). Beta actin was used as the housekeeping gene where the normalized expression ratio of the target genes was calculated using the 2^-∆∆Ct^ (Livak method) [14]. All reactions were carried out in triplicate from three independent experiments.

### 2.7. Global DNA Methylation and Hydroxymethylation Analysis

Global DNA methylation and hydroxymethylation levels were measures using 5mC DNA and Quest 5hmC DNA ELISA Kits, respectively (Zymo Research, CA, USA). DNA was denatured at 98 °C for 5 min and then an input of 100 ng per treatment condition was used to run the assay per the manufacturer’s protocol. Briefly, the denatured DNA was incubated in the plate for 1 h at 37 °C followed by several washes and incubation with a mixture of the primary antibody (anti-5mC or anti-5hmC) and the secondary antibody for 1 h at 37 °C. Finally, HRP developer was added to each well, color development was allowed for 10–60 min at room temperature, and absorbance was measured at 405–450 nm using a multimode plate reader (M3 Molecular Devices, San Jose, CA, USA). The percentages of 5mC and 5hmC in each sample were calculated using the prediction equation generated by logarithmically plotting the absorbance values of the corresponding standard curve. A standard curve with known percentages of 5 mC was generated by mixing the supplied negative controls (0% methylation) and positive controls (100% methylation) in certain ratios recommended in the manufacturer’s protocol. For the 5hmC standard curve, five supplied control DNA sets with 5hmC percentages that ranged from 0% to 55% were used. All samples were run in triplicate and the data represent the average from three different experiments.

### 2.8. DNA Bisulfite Modification and Methylation-Specific PCR

Genomic DNA was extracted from cells using DNeasy Blood & Tissue Kits (Qiagen) and submitted to bisulfite modification using the EpiTect Bisulfite Kit specific protocol (Qiagen). Bisulfite treatment of DNA deaminates the unmethylated cytosine residues and converts them to uracil but leaves the methylated cytosine residues unchanged. Therefore, bisulfite treatment changes DNA promoter sequence depending on the methylation status of cytosine residues. DNA recovery was assessed using NanoDrop™ One Microvolume UV-Vis Spectrophotometer (Thermo Fisher Scientific) to compare before bisulfite treatment and after bisulfite treatment DNA quantity. Assessment of DNA fragmentation was performed by RT-PCR where different primer pairs were developed to amplify ACTB (β-actin) gene amplicons with lengths varying from 209 to 718 bp—a method that was previously published by Ehrich et al. [15]. Modified DNA was then subjected to real-time PCR using methylated primers (anneal to methylated DNA) and unmethylated primers (anneal to unmethylated DNA) for leptin, IL1β, IL6, IL8, IL17, CXCL5, macrophage migration inhibitory factor (MIF), VEGF, TNF-α, and IFNγ that were designed using the Methprimer software (http://www.urogene.org/cgi-bin/methprimer/methprimer.cgi) (Table 2). The regions of interest in the promoter were chosen according to in silico analyses using UCSC database (http://genome.ucsc.edu) for CpG island prediction. Primers were designed using the following criteria for optimum MSP primer selection: CpG Island size > 100 bp, GC Percent > 50.0, and Observed/Expected CpGs > 0.60.

### 2.9. Western Blotting

Total protein was isolated using RIPA lysis buffer that consists of 20 mM Tris-HCl (pH 7.5), 150 mM NaCl, 1 mM Na2 EDTA, 1 mM EGTA,1% NP-40, 1% sodium deoxycholate, 2.5 mM sodium pyrophosphate, 1 mM b-glycerophosphate, 1 mM Na3VO4, and 1 µg/mL leupeptin (Cell Signaling, Danvers, MA, USA) supplemented with protease and phosphatase inhibitor cocktail (MS-SAFE) from Sigma-Aldrich. Nuclear protein was isolated using a specific Nuclear Extraction Kit (Active Motif, Carlsbad, CA, USA). Briefly, cells were suspended in 500 µL of 1X hypotonic buffer and incubated on ice for 15 min, after which, 25 µL of detergent was added and cells were vortexed for 10 s at a high speed. Cell suspension was then centrifuged for 30 s at 14,000× *g* in a prechilled microcentrifuge. The supernatant that contained the nuclear protein fraction was stored at −80° C until the time of analysis. Protein concentration was measured using the Pierce BCA Protein Assay Kit (Thermo Fisher Scientific). Proteins (10 μg) were resolved by 4%–12% Bis-Tris gradient gels (Bio-Rad, Des Plaines, IL, USA) and transferred to PVDF membranes. Membranes were blocked and incubated with the primary antibodies, HIF1α (H1alpha67 (ab1)) mouse monoclonal antibody and TET1 rabbit polyclonal antibody (ab191698) from Abcam, DNMT1 (H-300; sc-20701) rabbit polyclonal, DNMT3a (H-295; sc-20703) rabbit polyclonal and DNMT3b (N-19; sc-10235) goat polyclonal antibodies from SantaCruz Biotechnology (SantaCruz, CA, USA), overnight at 4 °C and then with infrared IRDye^®^—labeled secondary antibodies (LI-COR Biosciences, Lincoln, NE, USA) for 1 h at room temperature, protected from light. Membranes were washed with TBS + 0.1% Tween-20, dried for 1 h at room temperature and then scanned in the appropriate channel (700 nm for IRDye680TM antibodies, 800 nm for IRDye800TM antibodies) using an Odyssey Clx infrared imaging system. β actin mouse monoclonal antibody (Cell Signaling) was used as a loading control. Images were then quantified using Image Studio ver. 4.0 (LI-COR Biosciences, Lincoln, NE, USA) to calculate the intensity of the bands of the protein of interest relative to the expression of β actin from three independent experiments.

### 2.10. Measurement of DNMT Activity

Nuclear fraction of cellular protein was extracted using the EpiQuik Nuclear Extraction Kit (Epigentek, Farmingdale, NY, USA) and following the manufacturer protocol. Briefly, cells were washed with 1X phosphate buffer saline (PBS) and resuspended in the supplemented nuclear extraction buffer after adding dithiothreitol (DTT) and protease inhibitor cocktail at a 1:1000 dilution. Cell suspension was then incubated on ice for 10 min, vortexed vigorously for 10 s then centrifuged for 1 min at 12,000 rpm. The cytoplasmic extract was carefully removed from the nuclear pellet. DNMT activity was then assessed in the nuclear extract using a colorimetric EpiQuik DNMT activity assay kit (Epigentek) following the manufacture’s instruction. Briefly, nuclear protein fraction was incubated with SAM (s-adenosylmethionine) and DNMT substrate for 1 h. This was followed by incubation with the capture antibody, detection antibody, and the developing solution, sequentially. SAM plus substrate in absence of nuclear extract was used as a negative control and a mixture of SAM, substrate, and purified DNMT enzyme preparation supplied in the kit was used as a positive control. Absorbance was measured via a multimode microplate reader at 450 nm.

### 2.11. Chromatin Immunoprecipitation Assay (ChIP)

DNA and proteins in cultured cells were cross-linked via 1% formaldehyde at 37 °C for 15 min followed by washing and isolating cells in ice-cold PBS. The pellet was then lysed in SDS-lysis buffer (50 mM Tris–HCl pH 8.1, 10 mM EDTA, 1% SDS) supplemented with protease inhibitors, and sonicated for 20 cycles (10 s pulse and 30 s rest on ice) using Q500 Sonicator (Qsonica, Atkinson, NH, USA). The length of DNA fragments ranged from 400 to 600 base pairs as were verified by agarose gel electrophoresis. Sheared chromatin was diluted in a ChIP dilution buffer (0.01% SDS, 1.1% Triton X-100, 1.2 mM EDTA, 16.7 mM Tris-HCl, pH 8.1, 167 mM NaCl) (Millipore Sigma, Billerica, MA, USA). The chromatin was then immunoprecipitated via Dynabeads M-280 coated with normal mouse IgG or HIF1α antibodies overnight at 4 °C. Protein–DNA complexes were eluted and reverse cross-linked by heating at 65 °C for 6 h. The immunoprecipitation of HIF1α was confirmed by western blotting. DNA was purified using DNA purification kit (Qiagen). DNA samples were analyzed by real-time PCR as described above. TET1 primers were designed to produce an amplicon that encompass the region from −264 to −436 upstream to TET1 transcription start site (TSS). Normal mouse IgG was used as a negative antibody control. Primers, which amplify regions containing or lacking the HRE of PDK1 (pyruvate dehydrogenase kinase 1), a previously verified target of HIF1α [16], were used as positive and negative controls for HIF1α ChIP, respectively. All primers are listed in Table 1.

### 2.12. Statistical Analysis

The data were analyzed using Student’s t -test, or one-way ANOVA followed by a post-hoc test as appropriate. Data represent mean ± standard deviation (SD), and statistical significance was achieved if *p* < 0.05.

## 3. Results

### 3.1. Description of Experimental Models

The level of adipogenicity in differentiated adipocytes was measured by means of a visual grading system (grades 1 through 4) [13] using Oil Red O stain on days 0, 6, and 14 after cell incubation with differentiation-inducing media (Figure 1A). The relative percentage of cells at each grade was calculated on days 0, 6, and 14 (Figure 1B). As seen in Figure 1A,B, differentiation began as a gradual accumulation of fat in the cytoplasm of preadipocytes and progressed to higher adipogenicity grades; 65% of cells were at grades 3 and 4 by differentiation day 14 (Figure 1B). Adiponectin gene (AdipoQ) and the late adipogenesis marker, FABP4 (fatty acid binding protein 4), have been previously shown to closely reflect adipogenesis in vitro [17,18]. Accordingly, AdipoQ and FABP4 mRNA levels were next determined in differentiated adipocytes. Our results demonstrated significant increases in AdipoQ (3-fold by day 6 and 8.6-fold by day 14) and FABP4 (2-fold by day 6 and 6-fold by day 14) (Figure 1C).

To induce HIF1α production, adipocytes were cultured under hypoxia (2% O_2_) plus the HIF1α stabilizer, (DMOG, 500 μM) as previously described [19] and compared to control cells cultured under normoxia (20% O_2_) for 24 h. To inhibit HIF1α transcriptional activity, methyl 3-[[2-[4-(2-adamantyl) phenoxy] acetyl] amino]-4-hydroxybenzoate (CAY10585) was used [20]. We tested three different concentrations of CAY10585 (10, 20, and 30μM), which decreased the nuclear fraction of HIF1α protein, by 55%, 74%, and 90%, respectively (Figure 1D). CAY10585 concentration, that reduced HIF1α nuclear fraction by 90%, was used in the rest of experiments. To downregulate TET1 expression, we used siRNAs pool that consists of three target-specific 19–25 nt siRNAs designed for effective knockdown of the TET1 gene. The validation of TET1 silencing was performed by western blot analysis of the total protein that was collected from transfected cells. Figure 1E demonstrates a 90% reduction in TET1 protein in response to TET1-specific siRNAs.

### 3.2. Effect of Hypoxia on TET1 Expression in Differentiated Adipocytes

**Figure 2:**
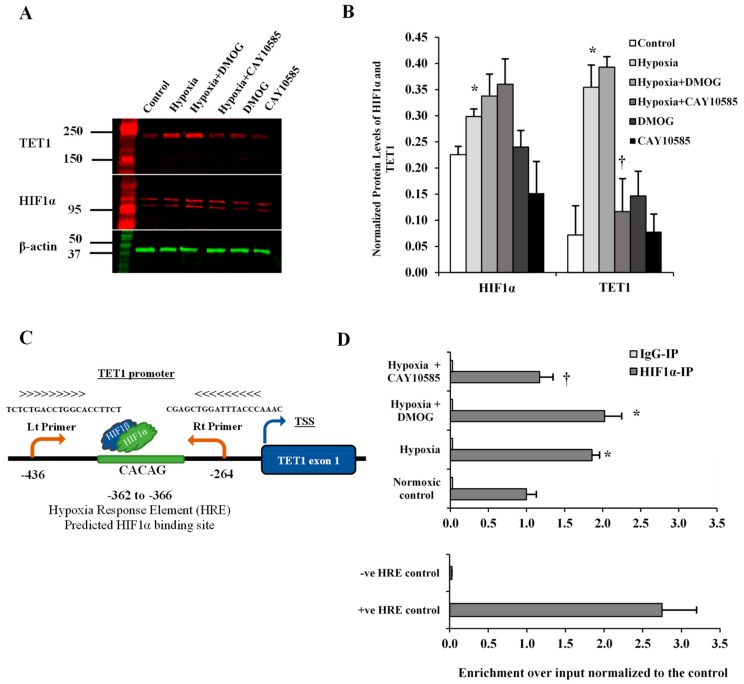
Effect of hypoxia on HIF1α and TET1 expression in differentiated adipocytes. (**A**,**B**) Western blot analysis and quantification of the normalized signal intensity of HIF1α and TET1 proteins in response to 24-h hypoxia with either a HIF1α stabilizer (DMOG) or a HIF1α inhibitor (CAY10585). (**C**) Illustration that demonstrates the predicted HIF1α binding site and the location of TET1 primers in the promoter of TET1 gene. (**D**) ChIP-qPCR using a specific antibody against HIF1α to analyze the presence of HIF1α protein at the promoter of TET1 gene. ChIP was performed in primary differentiated adipocytes cultured under normal or hypoxic conditions. Normal mouse IgG was used as a negative antibody control. primers, which amplify regions containing and lacking the HRE of PDK1 were used as positive and negative locus control, respectively. Results represent the means ± SD for three independent experiments in triplicates for each treatment (n = 9). (* *p* < 0.05) for comparison with control. (^†^ *p* < 0.05) for comparison with hypoxia.

HIF1α is a transcription factor that has been shown to induce TET1 transcription in cancer cells [21]. Also, bioinformatics studies showed that TET1 gene has a hypoxia-response element in its promoter which exists in all the hypoxia-inducible genes and to which HIF1α binds and induces gene transcription [22]. Therefore, we sought to investigate the effect of HIF1α induction on TET1 expression in differentiated adipocytes. To induce HIF1α production, adipocytes were cultured under hypoxia (2% O_2_) plus the HIF1α stabilizer, DMOG (500 μM) and compared to control cells cultured under normoxia (20% O_2_) for 24 hrs followed by measuring protein levels of HIF1α and TET1. Also, HIF1α protein binding to TET1 gene was measured via ChIP assay. Our data show that HIF1α and TET1 proteins increased by 12 folds and 3.3 folds, respectively (* *p* < 0.01) under conditions of hypoxia and HIF1α stabilization. The inhibition of HIF1α transcriptional activity, via CAY10585 (30 μM), suppressed the hypoxia-induced TET1 protein expression by 58% with a minimal effect on HIF1α protein levels (Figure 2A,B). Next, we examined whether HIF1α protein was attached to the promoter region of TET1 and whether the hypoxic conditions and HIF1α stabilization versus inhibition changed HIF1α occupancy in this region. A putative HIF1α binding site was predicted between −362 to −366 upstream to TET1 transcription start site (TSS). TET1 primers were designed to produce an amplicon that encompass the predicted HIF1α binding site (Figure 2C). We observed a significant enrichment (an increase by 83%) of TET1 promoter in ChIP assays using specific antibody for HIF1α (Figure 1D). This effect was significantly abrogated by the HIF1α inhibitor, CAY10585. No significant differences were observed when DMOG or CAY10585 were applied in the absence of hypoxia.

### 3.3. Effect of Hypoxia and HIF1α/TET1 Pathway on Global DNA Methylation and Hydroxymethylation in Differentiated Adipocytes

**Figure 3:**
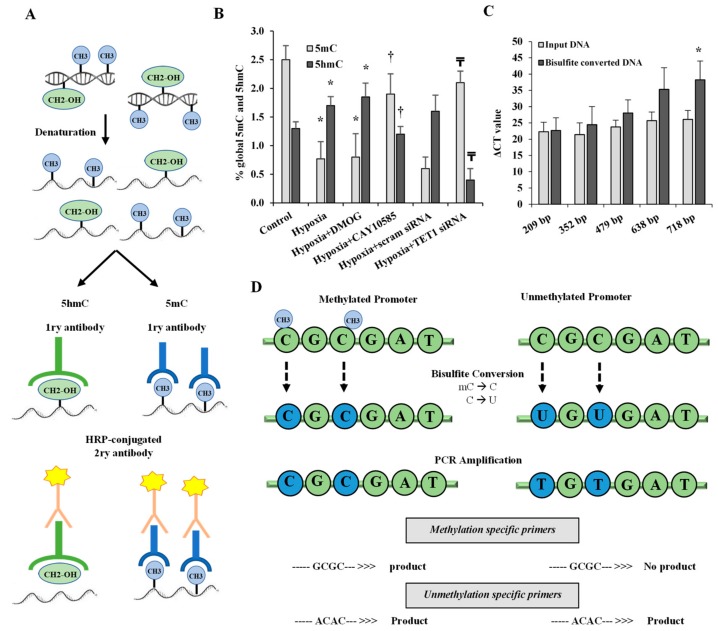
Effect of hypoxia on global DNA methylation and hydroxymethylation in differentiated adipocytes. (**A**) Illustration of the workflow for the 5 mC DNA and Quest 5hmC DNA ELISA Kits that were used to measure global DNA methylation and hydroxymethylation levels, respectively. (**B**) The percentage of global 5mC and 5hmC in primary differentiated adipocytes cultured under conditions of hypoxia ± DMOG, CAY10585, or silenced TET1 compared to standard culturing conditions (control). (**C**) To assess the quality of bisulfite converted DNA, ΔCT values were compared between bisulfite converted and input DNA for the amplification of several β-actin amplicons that ranged from 209 to 718 bp. (**D**) Outline of bisulfite conversion of genomic DNA where cytosine (**C**) is deaminated and converted to uracil (U) while methylated cytosine (mC) is protected and maintained as cytosine. Methylation-specific and unmethylation-specific primers are designed to detect methylated vs. unmethylated DNA, respectively. Results represent the means ± SD for three independent experiments in triplicates for each treatment (n = 9). (* *p* < 0.05) for comparison with control and (^†^ *p* < 0.05) for comparison with hypoxia and (^₸^ *p* < 0.05) for comparing hypoxia + TET1 siRNA and hypoxia + scram siRNA.

We measured global 5-mC and 5-hmC in differentiated adipocytes under conditions of hypoxia (2% O_2_) plus the HIF1α stabilizer, DMOG (500 μM) for 24 h or standard conditions (20% O_2_). Figure 3A illustrates the workflow for the 5mC DNA and Quest 5hmC DNA ELISA Kits that were used to measure global DNA methylation and hydroxymethylation levels, respectively. The results showed a decrease in the methylated (5mC) promoter fraction while 5-hmC levels increased significantly in adipocytes that were cultured under hypoxia compared to standard conditions. In contrast, silencing TET1 or inhibiting HIF1α significantly increased 5-mC and reduced 5hmC in hypoxic adipocytes (Figure 3B). No significant differences were observed when DMOG or CAY10585 were applied in the absence of hypoxia.

### 3.4. Effect of Hypoxia and HIF1α/TET1 Pathway on Adipocytokine Gene Expression and Promoter Methylation in Differentiated Adipocytes

**Figure 4:**
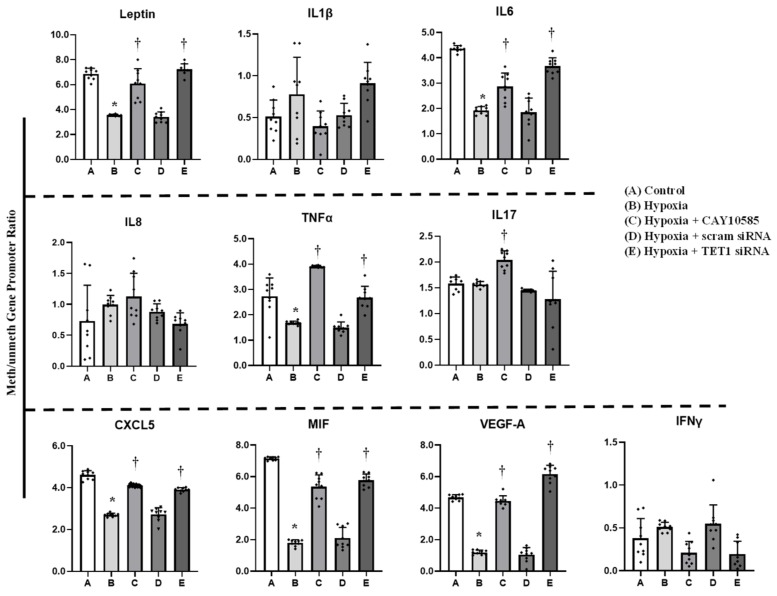
Effect of hypoxia and HIF1α/TET1 pathway on adipocytokine promoter methylation. Methylation-specific PCR analysis of the methylated/unmethylated ratios of leptin, IL1β, IL6, IL8, TNFα, IL17, CXCL5, MIF, VEGFA, and IFNγ in adipocytes that were cultured for 24 h under conditions of hypoxia ± CAY10585 or silenced TET1 compared to standard culturing conditions (control). Results represent the means ± SD for three independent experiments in triplicates for each treatment (n = 9). (* *p* < 0.05) for comparison with control and (^†^ *p* < 0.05) for comparison with hypoxia.

**Figure 5:**
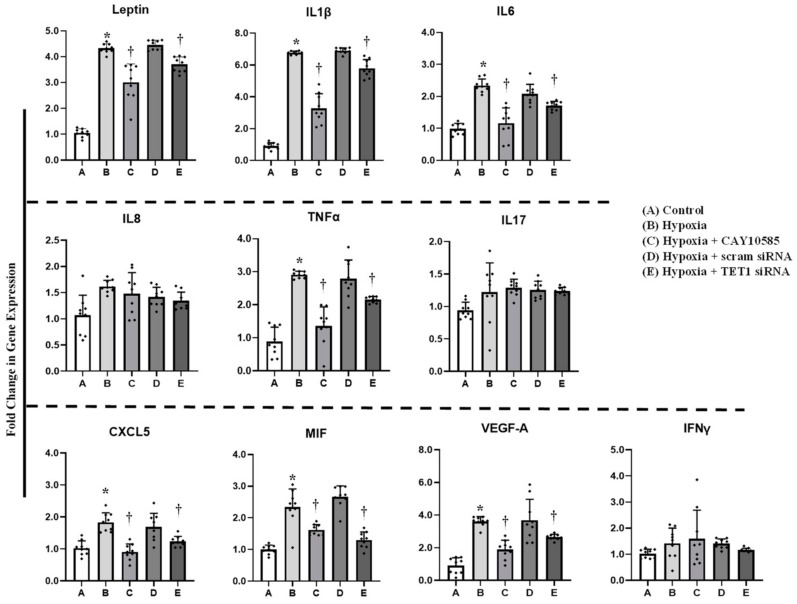
Effect of hypoxia and HIF1α/TET1 pathway on adipocytokine gene expression. Quantitative assessment by real-time PCR of mRNA levels of leptin, IL1β, IL6, IL8, TNFα, IL17, CXCL5, MIF, VEGFA, and IFNγ normalized to the house keeping gene, β-actin. Primary adipocytes were cultured for 24 h under conditions of hypoxia ± CAY10585 or silenced TET1 compared to standard culturing conditions (control). Results represent the means ± SD for three independent experiments in triplicates for each treatment (n = 9). (* *p* < 0.05) for comparison with control and (^†^ *p* < 0.05) for comparison with hypoxia.

Gene expression and promoter methylation were analyzed in a panel of ten inflammatory genes, leptin, IL1β, IL6, IL8, IL17, CXCL5, MIF, VEGF, TNF-α, and IFNγ. The absolute concentration of the DNA samples was measured on a NanoDrop™ UV-Vis Spectrophotometer before and after bisulfite treatment. The starting DNA concentration was on average 356 ng/μL. The bisulfite converted DNA recovery ranged from 56% to 61% of the input DNA. To assess DNA fragmentation after bisulfite treatment, we compared ΔCT values between bisulfite converted and input DNA for the amplification of several β-actin amplicons that ranged from 209 to 718 bp. We observed comparable CT values for the amplicons up to 638 bp which are well above the targeted amplicons in the current study that ranged from 111 to 241 bp. Bisulfite converted DNA was then subjected to Real-Time PCR using methylated and unmethylated primers for leptin, IL1β, IL6, IL8, IL17, CXCL5, MIF, VEGF, TNF-α, and IFNγ. Our data show that ratios of methylated to unmethylated fractions of gene promoters were lower under the hypoxic conditions compared to the control states (Figure 4) in all the tested genes except IL1β, IL8, IL17 and IFNγ. These reductions were significantly obtained in VEGF-A (−7%), MIF (−73%), IL6 (−55%), leptin (−50%), and CXCL5 (−41%). Methylation patterns were restored after HIF1α inhibition and TET1 silencing. We then proceeded by measuring the effect of hypoxia on adipocytokine gene expression. Hypoxia plus HIF1α stabilization induced mRNA levels of all the analyzed cytokines except IL8, IL17 and IFNγ (Figure 5). On the top of gene list that were induced in response to hypoxia are IL1β (6.8-fold) and leptin (4.4-fold), followed by VEGF-A (3.5-fold) and TNF-α (3-fold). Inhibiting HIF1α via CAY10585 suppressed the hypoxia-induced increases in the mRNA levels of these genes. To confirm the role of TET1 in adipocytokine expression, TET1 expression was suppressed using specific siRNA as previously described [23]. Silencing TET1 interfered with changes in adipocytokine gene expression in response to hypoxia and HIF1α stabilization except for the IL1β gene. Induction in gene expression mirror-imaged the reductions in promoter methylation except in IL1β where no changes in DNA methylation were observed. No significant differences were observed when DMOG or CAY10585 were applied in the absence of hypoxia. Adding DMOG to hypoxic cells enhanced the effect of hypoxia; however, results were not statistically different from hypoxia alone. This phenotype could be attributed to the dual role of hypoxia that effectively induces both HIF1α gene expression and protein stabilization. Accordingly, data for hypoxia +DMOG treatment were redacted from Figure 4 and Figure 5 to avoid redundancy.

### 3.5. Evaluation of the Role of DNMT in Hypoxia-Induced Adipocytokine Hypomethylation

**Figure 6:**
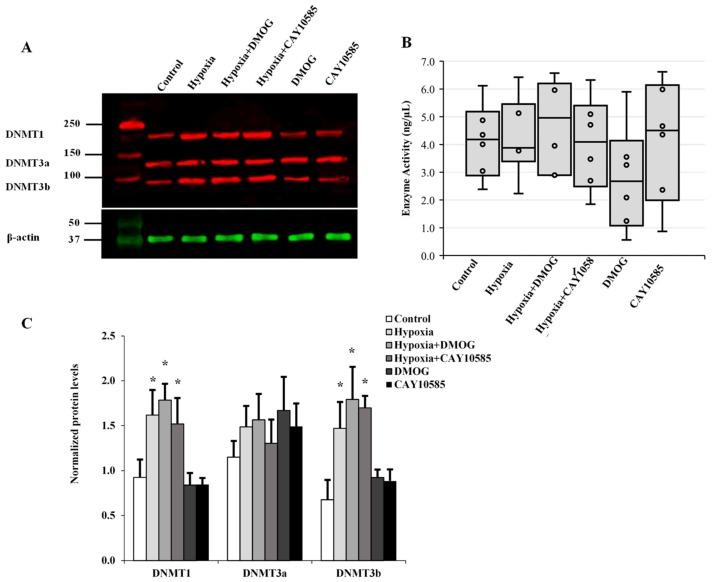
The role of DNMTs in hypoxia-induced adipocytokine hypomethylation in differentiated adipocytes. (**A**) Western blot analysis and quantification of the normalized signal intensity of DNMT1, DNMT3a, and DNMT3b (**C**) and DNMT enzymatic activity (**B**) in adipocytes cultured under hypoxic and normoxic conditions for 24 h. Results represent the means ± SD for three independent experiments in triplicates for each treatment (n = 9). (* *p* < 0.05) for comparison with control.

The transfer of methyl groups to DNA is mediated by a group of enzymes known as DNMTs. Currently, three DNMTs have been identified in mammalian cells, namely DNMT1, DNMT3a, and DNMT3b. Thus, in addition to active demethylation, DNA hypomethylation might be achieved via inhibition of DNMT enzyme activity or reduction in its cellular protein levels. In order to assess any potential contribution of the DNMTs to the observed hypoxia-induced global and gene-specific DNA hypomethylation, proteins collected from hypoxic adipocytes were analyzed for DNMT protein expression (Figure 6A) and activity (Figure 6B). Our data reveal that protein levels of DNMT1 and DNMT3b increased by 84% and 64%, respectively, compared to the control. However, this increase was borderline significant (*p* = 0.045). No changes in DNMT3a protein levels or DNMT enzyme activity were detected in adipocytes under hypoxic conditions.

## 4. Discussion

In the current study, the main findings are that (1) gene expression of leptin and inflammatory cytokines was induced in adipocytes cultured under hypoxic conditions; (2) this response was mediated by DNA hypomethylation in the corresponding gene promoters; (3) HIF1α and the DNA hydroxymethylase TET1 contributed, at least in part, to the hypoxia-induced gene promoter hypomethylation.

Previous studies have suggested an association between hypoxia and inflammation [24]. The latter is a hallmark of obesity and is thought to be the underlying mechanism of several obesity-associated comorbidities such as metabolic and cardiovascular diseases [25,26]. The recognition of adipose tissue ability to secrete inflammatory cytokines has advanced our understanding of obesity-associated inflammation. As the mass of the adipose tissues expands, hypoxia develops and contributes to the generation of inflammation in adipocytes and adjacent tissues [27]. In the current study, we used an in vitro hypoxic adipocyte culture model to study human adipose tissue inflammatory responses to hypoxia and identify DNA methylation as an intermediate pathway in this link. Our results implicate HIF1α and TET1 as key players in the regulation of hypoxia-induced inflammation.

One hypothesis is that obesity-associated induction of inflammatory cytokines by adipocytes is mediated by an epigenetic mechanism that is sensitive and responsive to changes in the tissue microenvironment, mainly oxygen supply. The role of hypoxia in inducing inflammatory cell infiltration in adipose tissues has been reported in mice and suggested to be partly dependent on HIF1α [28,29]. However, the intermediate pathways between hypoxia and inflammation in adipose tissues remain to be characterized. The HIF1α gene is constitutively expressed under normoxic conditions; however, its stability and transcriptional activity increase under hypoxia [30]. As a transcription factor, HIF-1α plays an important role in cellular response to low oxygen levels in mammals by regulating the transcription of genes involved in cell proliferation and survival, angiogenesis, vascularization, immune response, and metabolism [31]. This response maintains cellular homeostasis, yet dysregulation of the HIF1α pathway and its downstream signaling have been reported in several pathogenic conditions that are characterized by hypoxia ranging from infection to cancer.

It is now generally accepted that white adipocytes are responsible for secreting a major part of the circulating adipocytokines, which links obesity to systemic inflammation and related co-morbidities. Several studies have determined hypoxia as a primary characteristic of adipose tissues in obesity. Hypoxia in obesity occurs when oxygen availability does not match the demand of the expanding adipose tissue, resulting in decreased oxygen tension [32]. HIF-1α is considered the main mediator of cellular responses to hypoxia [30]. Acting as a transcription factor, HIF-1α has been shown to induce the transcription of several genes that are involved in inflammation such as leptin, matrix metalloproteinases (MMP2/9), and adipocytokines [33,34,35].

High levels of hypoxia demonstrated positive correlations with DNA global hypomethylation in cancer and other hypoxic conditions [36]. Furthermore, hypoxia is suggested to modify methylation of specific genes and one of the most common examples is erythropoietin that exhibits hypomethylation and overexpression under conditions of chronic hypoxia such as living at a high altitude [37]. The DNA hydroxymethylase TET1 has been reported to be transcriptionally activated by HIF-1α in cancer cells [38,39]. Mariani et al. [21] demonstrated that 48 h of hypoxia Increased TET1 expression and accumulation of 5-hmC in different cancer cell lines. Also, Laukka et al. [40] reported that TET enzymes, specially TET1, retain high activity under hypoxic conditions in cancer cells. Hypoxia has been also shown to induce the expression of TET enzymes in hepatoblastoma cells [22], breast cancer cells [39], and other types of cancer [41]. Despite accumulating evidence of the contribution of TET enzymes in hypoxia-induced hypomethylation, this role has only been documented in the context of cancer, and similar findings have not been demonstrated under hypoxic conditions in non-cancerous cells such as adipocytes that are high likely to be hypoxic in obese people.

In the current study, we exposed human, differentiated adipocytes to hypoxia, explored modifications in global and adipokine DNA methylation, and confirmed the contribution of TET1 enzyme to the outcome. Our data demonstrate an increase in protein levels of HIF1α in adipocytes that were cultured under hypoxic conditions. This is consistent with previous studies that suggested a role of hypoxia in stabilizing HIFα. These studies showed HIF1α to be constitutively expressed in all cells; yet, in the presence of oxygen, the α subunit of HIF1is hydroxylated via the oxygen-dependent enzyme, prolyl hydroxylase [42,43]. In a hypoxic environment, the hydroxylation of the α subunit is diminished and it binds to the β subunit yielding the active form of HIF-1α [44].

In our study, the induction in HIF1α was associated with increases in TET1 protein levels and global DNA hydroxymethylation and decreases in global DNA methylation and the methylated fraction of leptin and adipocytokines. These responses were abolished by the HIF1α transcriptional activity inhibitor, CAY10585, indicating a role of HIF1α in the hypoxia-induced DNA hypomethylation and subsequent induction of adipocytokine expression. These data are supported by previous studies that identified HIF1α as a key player in adipocyte inflammation. These studies showed that HIF1α knocked down mice resisted the development of adipose inflammation and insulin resistance that were induced by high fat diet compared to their wild-type littermates [45,46].

In addition to HIF1α-dependent pathways, hypoxia induces HIF1α-independent inflammation via stimulating lipolysis and consequently increases free fatty acids, downregulating peroxisome proliferator-activated receptor gamma (PPARγ) expression, and increasing the expression of macrophage inflammatory protein-1α (MIP-1α) and macrophage infiltration [34]. The most common HIF1α-dependent pathways that were frequently discussed in the literature are related to the cross talk between HIF1α and NFkB—the latter is a transcriptional factor that mediates the production of several proinflammatory cytokines [47].

HIF1α is capable of activating the transcription of several chemokines and chemokine receptors such as CCL5 and CXCL12 [48]. HIF1α has been also shown to transactivate the human leptin gene promoter in the placental tissue from preeclamptic women [49]. The role of HIF1α in inducing cytokine production has been shown in a few reports, such as that of Jeong et al. [50] whereby HIF1α inhibition abrogated the deferoxamine-induced cytokine production (IL6, IL8 and TNFα) in human mast cells, HMC-1 cells. Further, Budda et al. [51] demonstrated that HIF-1α controlled IL-22 expression in CD4 T Cells under hypoxia. However, the exact mechanism by which HIF1α controls the production of these inflammatory mediators was not completely understood. To the best of our knowledge, this is the first study to examine the epigenetic consequences of HIF1α induction in adipocytes as a mechanism of induced inflammation under conditions of hypoxia.

Recent studies showed that TET enzymes convert 5-mC to 5-hmC which is modified through several suggested mechanisms, including deamination and decarboxylation, that ultimately lead to base excision repair and replacement with an unmethylated cytosine [10]. This TET-mediated mechanism has been thought to contribute to aberrant oncogenic expression in some cancers [12]. Nevertheless, its contribution in inflammation has been suggested only recently by Sun et al. [52] where they reported a contribution of TET1 to the activation of macrophages via inducing 5-hydroxymethylation in the promoter of TNFα and other pro-inflammatory cytokines.

Epigenome-wide association studies have reported global DNA hypomethylation and the differential methylation of some inflammatory and adipogenesis genes in peripheral blood cells of obese individuals compared with their lean counterparts [53,54,55]. Yet, it is critical to analyze tissue-specific DNA methylation profiles instead of testing easily accessible blood samples especially after differential DNA methylation patterns were noted among various tissues in previous studies [56]. Also, it is important to dissect the underlying mechanisms that contribute to the differential global and gene-specific methylation in obesity or other morbidities so that they can be targeted by preventive and therapeutic modalities. In the current study, we explored the role of TET1 enzyme in hypoxia-induced hypomethylation using primary adipocytes, hoping to reveal the mechanism by which dysfunctional adipose tissues produce excess inflammatory cytokines in obesity and comorbidities. We also excluded the possibility that reductions in DNMT expression or activity have contributed to the observed DNA hypomethylation in hypoxic adipocytes. In fact, studies that reported the effect of hypoxia on DNMTs have shown inconsistent findings where either DNMT augmentation [7,57] or suppression [8] were observed.

DNMTs are multi-domain proteins that contain a catalytic C-terminal domain and a regulatory N-terminal domain [58]. Previous studies have shown that DNMT activity is controlled by the interaction of both domains leading to an allosteric activation of the enzyme [59]. Moreover, recent structural and biochemical data demonstrate that posttranslational modifications, including phosphorylation, methylation, ubiquitination, acetylation, and sumoylation, were shown to exert regulatory function on DNMT activity, specificity and localization [60]. Finally, DNMTs were also shown to be regulated by a myriad of interacting protein partners that allosterically regulate their catalytic activity [61]. In the current study, we observed an induction of DNMT1 and DNMT3b protein levels, yet there was no increase in DNMT activity. It is possible that under conditions of oxidative stress, various structural, biochemical, or oxidative posttranslational modifications may influence DNMT domain arrangements and subsequently its biological activity. Findings from the current study could only exclude the role of DNMT in the observed DNA hypomethylation; however, they are not enough to understand the status of DNMT activity under hypoxic conditions. Therefore, further studies that investigate DNMT regulation and contribution to DNA methylation profiles in hypoxic microenvironments are warranted.

One major limitation of the current study is DNA fragmentation secondary to bisulfite conversion, an approach that is currently considered the gold standard for analyzing DNA methylation. Several commercial bisulfite conversion kits provide good conversion efficiencies; however, DNA loss and fragmentation remain a huge obstacle that hampers DNA methylation profiling assays. A study by Kint et al., [62] that compared 12 commercial bisulfite conversion kits reported that Epitech bisulfite kit from Qiagen had the best recovery and conversion efficiency and the least DNA fragmentation. Utilizing this kit, ΔCT values were not statistically significant between bisulfite converted and input DNA when DNA fragments as long as 467 bp were amplified. In our study, we used a similar approach to assess DNA quality after bisulfite treatment with Epitech kit. We compared ΔCT values between bisulfite converted and input DNA for the amplification of several β-actin amplicons that ranged from 209 to 718 bp. Our data demonstrated comparable ΔCT values for the amplicons up to 638 bp. Significant differences were only observed when the targeted amplicon was 718 bp which is much larger than the targeted amplicons in this study (111 to 241 bp)

## 5. Conclusions

Taken together, our study reveals a new epigenetic mechanism for the induced production of inflammatory cytokines in hypoxic adipocytes. This mechanism involves the hypoxia-driven transcription factor HIF1α and the DNA hydroxylase/demethylase enzyme TET1. We propose that some of the downstream candidates for this mechanism include leptin, IL1β, IL6, TNFα, CXCL5, MIF, and VEGFA. Yet, further studies are needed to comprehensively explore other genes that could be affected by this mechanism in adipocytes and other tissue targets.

## Figures and Tables

**Table 1:** Sequences of primers used for real-time PCR.

Gene Name	RefSeq Accession Number	Primer	Amplicon Size	Tm	Primer Sequence (5′-3′) Forward
leptin	NM_000230.3	Fw	150	60.0	GGCTTTGGCCCTATCTTTTC
		Rv		60.0	CCAAACCGGTGACTTTCTGT
IL1β	NM_000576.3	Fw	205	60.0	GGGCCTCAAGGAAAAGAATC
		Rv		60.0	TTCTGCTTGAGAGGTGCTGA
IL6	NM_000600.5	Fw	180	59.8	AGGAGACTTGCCTGGTGAAA
		Rv		59.8	CAGGGGTGGTTATTGCATCT
IL8	NM_000584.4	Fw	227	60.0	TAGCAAAATTGAGGCCAAGG
		Rv		60.0	AAACCAAGGCACAGTGGAAC
IL17	NM_002190.3	Fw	216	60.0	CCCCAGTTGATTGGAAGAAA
		Rv		60.0	GAGGACCTTTTGGGATTGGT
CXCL5	NM_002994.5	Fw	218	60.0	GACGGTGGAAACAAGGAAAA
		Rv		59.9	GCTTAAGCGGCAAACATAGG
MIF	NM_002415.2	Fw	234	59.6	AGAACCGCTCCTACAGCAAG
		Rv		59.8	ATTTCTCCCCACCAGAAGGT
VEGF	NM_001025366.3	Fw	186	60.0	CCCACTGAGGAGTCCAACAT
		Rv		60.0	TTTCTTGCGCTTTCGTTTTT
TNF-α	NM_000594.4	Fw	173	60.1	TCCTTCAGACACCCTCAACC
		Rv		60.0	AGGCCCCAGTTTGAATTCTT
IFNɣ	NM_000619.3	Fw	198	59.6	TCCCATGGGTTGTGTGTTTA
		Rv		59.7	AAGCACCAGGCATGAAATCT
TET1	NM_030625.3	Fw	173	59.8	AGGTCCAGGGCCAAATAACT
		Rv		59.9	AGAAGGTGCCAGGTCAGAGA
β-actin	NG_007992.1	Fw	209	59.9	AGAAAATCTGGCACCACACC
		Rv		59.9	AACGGCAGAAGAGAGAACCA
β-actin	NG_007992.1	Fw	352	60.0	AAACTGGAACGGTGAAGGTG
		Rv		59.9	CTCAAGTTGGGGGACAAAAA
β-actin	NG_007992.1	Fw	479	59.9	AAGATGACCCAGGTGAGTGG
		Rv		59.9	GGGGTGTTGAAGGTCTCAAA
β-actin	NG_007992.1	Fw	638	59.9	CTCTTCCAGCCTTCCTTCCT
		Rv		60.0	AAAGCCATGCCAATCTCATC
β-actin	NG_007992.1	Fw	718	59.0	CTCTTCCAGCCTTCCTTCCT
		Rv		60.0	CACCTTCACCGTTCCAGTTT
FABP4	NM_001442.3	Fw	181	60.0	TACTGGGCCAGGAATTTGAC
		Rv		60.0	GTGGAAGTGACGCCTTTCAT
AdipoQ	NM_001177800.2	Fw	173	60.0	CCTAAGGGAGACATCGGTGA
		Rv		60.0	GTAAAGCGAATGGGCATGTT
PDK1 (+ve)	NC_000002.12	Fw	100	62.0	CGCCCTGTCCTTGAGCC
		Rv		62.9	CGGTATGGAGCGTCCCCT
PDK1 (-ve)	NC_000002.12	Fw	486	56.5	CTGATAAGACTACACTGGACG3
		Rv		56.6	CCCAAGATACTACACTACCATC

PDK1 (+ve) and PDK1 (-ve) are positive and negative controls for HIF1α ChIP assay, respectively.

**Table 2:** Sequences of primers used for methylation-specific real-time PCR.

Gene Name	RefSeq Accession Number	Primer	Amplicon Size	Tm	GC%	Primer Sequence (5′-3′) Forward
leptin	NG_007450.1	Fw M	111	59.6	72.73	TAGGATTAACGAGGGCGTAGTC
		Rv M		58.7	62.50	AACCCCTTAAAAAAATACTTCGAA
		Fw U	113	57.4	72.00	TTAGGATTAATGAGGGTGTAGTTGT
		Rv U		59.0	64.00	CAACCCCTTAAAAAAATACTTCAAA
IL1β	NG_008851.1	Fw M	146	59.3	52.00	TAATTTTAATATTTTGGGAGGTCGA
		Rv M		58.4	68.00	CAACTAAAACTACAAACACCTACCG
		Fw U	144	57.4	52.00	TAATTTTAATATTTTGGGAGGTTGA
		Rv U		57.0	72.00	ACTAAAACTACAAACACCTACCACC
IL6	NG_011640	Fw M	223	59.8	65.22	GACGGATTATAGTGTACGGTTGC
		Rv M		59.2	52.17	ATAAAATCATCCATTCTTCACCG
		Fw U	223	58.6	68.00	GATGGATTATAGTGTATGGTTGTGG
		Rv U		59.1	48.00	ATAAAATCATCCATTCTTCACCAAT
IL8	NG_029889.1	Fw M	146	59.1	60.00	TTTTTGAGTAGTTGGGATTATAGGC
		Rv M		59.1	69.57	ACACTTTAAAAATCCGAAACGAA
		Fw U	149	57.9	61.54	TTTTTTGAGTAGTTGGGATTATAGGT
		Rv U		58.7	68.00	CAACACTTTAAAAATCCAAAACAAA
IL17	NG_033021.1	Fw M	138	59.1	54.55	TTTTTATGATTTTATTGGGGGC
		Rv M		55.3	44.00	ATAAACAAAATATAACGCTATCGTC
		Fw U	142	59.3	48.00	ATTTTTTTATGATTTTATTGGGGGT
		Rv U		50.5	46.15	AATAAACAAAATATAACACTATCATC
CXCL5	NC_000004.12	Fw M	186	56.9	56.00	TAATTTTCGTTTTTTTAATTTTCGT
		Rv M		58.8	76.00	GCTAACGATAAACCCTAACTACGTC
		Fw U	188	54.5	57.69	TTAATTTTTGTTTTTTTAATTTTTGT
		Rv U		54.8	76.92	CACTAACAATAAACCCTAACTACATC
MIF	NG_012099.1	Fw M	136	56.7	76.00	GGTTTATCGTCGTATTTTTATTTTC
		Rv M		57.0	60.87	CAACCTATTCTCCACTTAACGAC
		Fw U	137	54.9	76.92	GTTTATTGTTGTATTTTTATTTTTGG
		Rv U		59.8	60.00	TCCAACCTATTCTCCACTTAACAAC
VEGF	NG_008732.1	Fw M	236	58.8	66.67	TAGTTAGAGTCGGGGTGTGTAGAC
		Rv M		59.1	76.19	GAAAAACCGAACAAAAACGAA
		Fw U	241	58.7	68.00	TAGTTAGAGTTGGGGTGTGTAGATG
		Rv U		59.0	70.83	AAAACAAAAAACCAAACAAAAACA
TNF-α	NG_007462.1	Fw M	151	59.3	56.00	GAGTATTGAAAGTATGATTCGGGAC
		Rv M		59.1	68.00	CAACAAACAAAAAAACGTAATAACG
		Fw U	147	57.5	56.00	GTATTGAAAGTATGATTTGGGATGT
		Rv U		55.7	72.00	ACAAACAAAAAAACATAATAACACC
IFNγ	NG_015840.1	Fw M	194	55.0	46.15	TTTTGATTAATATAGTGAAATTTCGT
		Rv M		58.6	68.00	TCACCCAAACTAAAATACAATAACG
		Fw U	189	50.1	45.83	TTGATTAATATAGTGAAATTTTGT
		Rv U		55.3	70.83	CCCAAACTAAAATACAATAACACA
